# Correlation of nuclear pIGF-1R/IGF-1R and YAP/TAZ in a tissue microarray with outcomes in osteosarcoma patients

**DOI:** 10.18632/oncotarget.28215

**Published:** 2022-03-09

**Authors:** Eric R. Molina, Letitia K. Chim, Salah-Eddine Lamhamedi-Cherradi, Sana Mohiuddin, David McCall, Branko Cuglievan, Sandhya Krishnan, Robert W. Porter, Davis R. Ingram, Wei-Lien Wang, Alexander J. Lazar, David W. Scott, Danh D. Truong, Najat C. Daw, Joseph A. Ludwig, Antonios G. Mikos

**Affiliations:** ^1^Department of Sarcoma Medical Oncology, Division of Cancer Medicine, The University of Texas, MD Anderson Cancer Center, Houston, TX, USA; ^2^Department of Bioengineering, Rice University, Houston, TX, USA; ^3^Division of Pediatrics, The University of Texas, MD Anderson Cancer Center, Houston, TX, USA; ^4^Department of Pathology, Division of Pathology and Laboratory Medicine, The University of Texas, MD Anderson Cancer Center, Houston, TX, USA; ^5^Department of Genomic Medicine, Division of Cancer Medicine, The University of Texas, MD Anderson Cancer Center, Houston, TX, USA; ^6^Department of Statistics, Rice University, Houston, TX, USA

**Keywords:** osteosarcoma, YAP/TAZ, IGF-1R, nuclear IGF-1R, mechanotransduction

## Abstract

Osteosarcoma (OS) is a genetically diverse bone cancer that lacks a consistent targetable mutation. Recent studies suggest the IGF/PI3K/mTOR pathway and YAP/TAZ paralogs regulate cell fate and proliferation in response to biomechanical cues within the tumor microenvironment. How this occurs and their implication upon osteosarcoma survival, remains poorly understood.

Here, we show that IGF-1R can translocate into the nucleus, where it may act as part of a transcription factor complex. To explore the relationship between YAP/TAZ and total and nuclear phosphorylated IGF-1R (pIGF-1R), we evaluated sequential tumor sections from a 37-patient tissue microarray by confocal microscopy. Next, we examined the relationship between stained markers, clinical disease characteristics, and patient outcomes. The nuclear to cytoplasmic ratios (N:C ratio) of YAP and TAZ strongly correlated with nuclear pIGF-1R (r = 0.522, *p* = 0.001 for each pair). Kaplan–Meier analyses indicated that nuclear pIGF-1R predicted poor overall survival, a finding confirmed in the Cox proportional hazards model.

Though additional investigation in a larger prospective study will be required to validate the prognostic accuracy of these markers, our results may have broad implications for the new class of YAP, TAZ, AXL, or TEAD inhibitors that have reached early phase clinical trials this year.

## INTRODUCTION

Osteosarcoma (OS), the most common primary tumor of the bone in the pediatric population and second most common in adults (following multiple myeloma), is characterized by a remarkable degree of intratumoral heterogeneity in cell phenotypes, gene expression, and chemoresistance [1–3]. The combination of multi-agent chemotherapy and improved surgical techniques have increased 5-year survival to 70% in patients diagnosed with localized disease.

In contrast, the survival rates for patients with recurrent disease or metastatic disease at diagnosis have barely changed, having remained around 20% for more than five decades [4]. Among the roadblocks that have stymied the advent of new therapies, perhaps the most impenetrable derives from the unique genetic etiology of OS. Whereas 10–15% of OS tumors exhibit relatively specific mutations in Rb or p53, non-targetable mutations in tumor suppressors associated with familial retinoblastoma [5, 6] and Li Fraumeni syndrome [7], most OSs originate as a byproduct of chromothripsis, the result of a catastrophic genomic event of unclear genesis [8–10]. The ensuing chromosomal and mutational diversity leaves oncologists without a singular target to direct their therapeutic focus. Despite their high mutation burden, OS has proven surprisingly recalcitrant to the numerous immunotherapies that have revolutionized the treatment of other mutation-high cancers.

Without OS-specific proteins to target, investigators have increasingly sought to explore whether oncogenic signaling cascades common in other cancers may also play a role in OS. The MAPK, IGF/PI3K/mTOR, and YAP/TAZ pathways, in particular, have generated much interest. Herein, we characterize a potential relationship in OS between the IGF-1R receptor tyrosine kinase (RTK) and two downstream mediators of the Hippo pathway, YAP and TAZ.

The IGF-1R/PI3K/mTOR cascade has been the subject of clinical trials in osteosarcoma with variable success [11–15]. Ligand-mediated receptor phosphorylation is known to initiate a signaling kinase cascade propagated through sequential activation of IRS-1, PI3K, and Akt, ultimately culminating in mTOR activation. Traditionally thought to function exclusively at the cell surface, recent data suggests that ligand-receptor engagement mediates IGF-1R endocytosis. Following its internalization, IGF-1R can recirculate back to the plasma membrane or transit the nuclear membrane where it joins transcription factors to exert complex epigenetic effects [16–18]. Significant controversy exists about the prognostic value of IGF-1R in its ability to predict response to downstream mediators PI3K and mTOR [18, 19].

Yes-associated protein 1 (YAP) and transcriptional coactivator with PDZ-binding motif (WWTR1, TAZ) are mechanoresponsive transcription factors phosphorylated by the active Hippo tumor suppressor pathway. Upon phosphorylation by MST1 and LATS (the mammalian orthologs of Hippo and Warts in Drosophila), YAP and TAZ (often referred to together as YAP/TAZ given their significant functional overlap) undergo binding to 14-3-3, which tags them for nuclear exclusion and degradation. Alternatively, if the Hippo pathway is inactive, YAP/TAZ remain unphosphorylated and translocate into the nucleus [20]. Studies in human mesenchymal stem cells (MSCs) have indicated that stiff microenvironments promote cell spreading, increase nuclear YAP and TAZ, and facilitate cell reprogramming towards an osteogenic lineage; conversely, cell confinement and less stiff microenvironments contribute to commitment towards an adipogenic lineage [21, 22]. Our lab and others have shown that YAP/TAZ localization is also influenced by the architecture and dimensionality of the tumor niche, highlighting the complexity of what cells ‘sense’ as their physical microenvironment [23, 24]. YAP/TAZ-mediated mechanotransduction has also been implicated in tumor chemoresistance and, potentially, worse OS survival [25–27].

In the present study, we evaluate the association between the IGF-1/mTOR and YAP/TAZ pathways, with a major emphasis on the nuclear (i.e., activated) state of phosphorylated IGF-1R (pIGF-1R), non-phosphorylated IGF-1R, and nuclear-to-cytoplasmic ratios of YAP and TAZ. This was accomplished using a human OS tissue microarray (TMA) comprised of 37 post-treatment OS tumor specimens. We performed confocal imaging of each TMA section and quantified nuclear and cytoplasmic protein intensity using semi-automated cell segmentation algorithms. Nuclear staining for pIGF-1R, total IGF-1R, and nuclear to cytoplasmic ratios (N:C ratios) for YAP/TAZ were correlated the patients’ clinical characteristics. Kaplan–Meier analyses were performed, and a proportional hazards model was used to estimate each parameter’s contribution to overall survival. Overall, this research sheds new light on the interrelationship between the IGF-1R/PI3K/mTOR and YAP/TAZ cancer-related pathways.

## RESULTS

### Patient demographics and biopsy characteristics

All patients received care at the University of Texas MD Anderson Cancer Center between November 1989 and December 2018. Biopsies were taken following neoadjuvant treatment from 37 patients. 67.6% of patients were male, and 37.8% were female (Table 1). Biopsies were taken from the primary tumor (27.0%), recurrent tumor (5.4%), or a metastatic site, most commonly the lung (67.6%). The mean age at diagnosis was 33.7 years, whereas the median age (interquartile range; total range) was 28.75 years (16.6–45.9; 10.4–79.5 years).

**Table 1:** Patient demographics and biopsy characteristics

		***n***	***Percentage***
*Race/Ethnicity*	Asian	2	5.4%
	Black or African American	4	10.8%
	White (Hispanic or Latino)	6	16.2%
	White (Not Hispanic or Latino)	25	67.6%
*Gender*	Female	14	37.8%
	Male	23	62.2%
*TMA Biopsy Site*	Primary	10	27.0%
	Metastatic: Other Site	4	10.8%
	Metastatic: Lung	21	56.8%
	Local Recurrence	2	5.4%

### Correlation of stained markers

**Figure 1:**
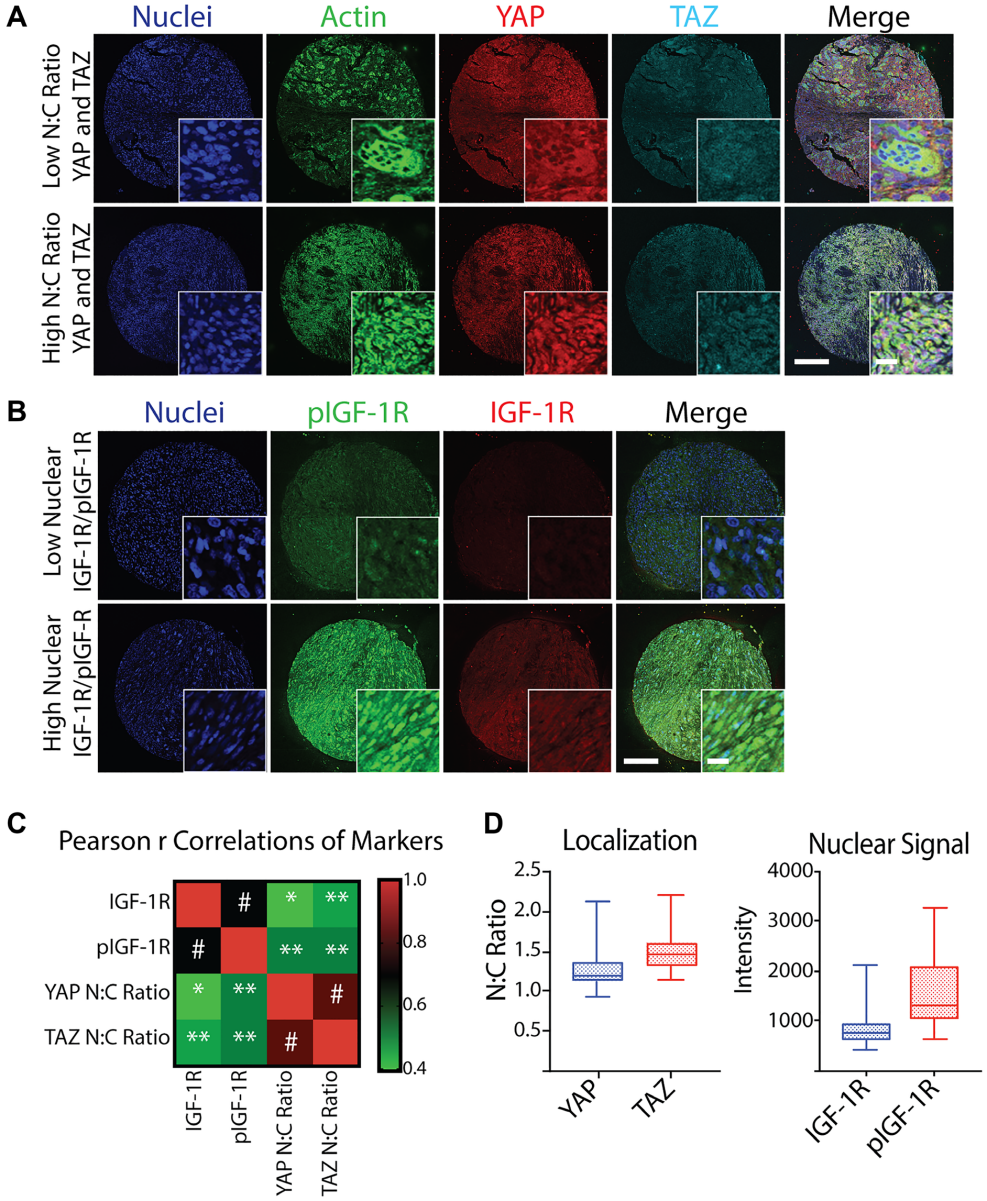
Quantification and correlation of YAP N:C ratio, TAZ N:C ratio, nuclear IGF-1R intensity, and nuclear pIGF-1R intensity from a TMA of post-treatment osteosarcoma biopsies. (**A**) Representative confocal microscopy images of osteosarcoma taken at post-treatment biopsy in a tissue microarray (TMA) displaying low (top) and high (bottom) N:C ratio of YAP and TAZ co-stained with Hoechst (nuclei, blue), phalloidin (actin, green), scale = 250 μm, inset scale = 20 μm. (**B**) Representative confocal microscopy images of osteosarcoma TMA displaying low (top) and high (bottom) nuclear IGF-1R and pIGF-1R (bottom) co-stained with Hoechst (nuclei, blue), scale = 250 μm. (**C**) Heatmap of Spearman r Correlations of YAP N:C ratio, TAZ N:C ratio, mean nuclear IGF-1R intensity, and mean nuclear pIGF-1R intensity for each patient represented in the TMA (*n* = 37 for IGF-1R/pIGF-1R and *n* = 36 for YAP/TAZ due to loss of one sample on TMA) with corresponding *p* values. ^*^ *p* < 0.05, ^**^ *p* < 0.01, ^#^ *p* ≤ 0.001. (**D**) Box plots of average marker values per patient across all patients represented in the TMA. Shaded boxes with inner line represent the interquartile range and median, where whiskers represent the range of calculated patient values.

YAP, TAZ, IGF-1R, and pIGF-1R demonstrated a wide range of localization and overall signal, which is expected given the high degree of morphological inter-patient and intra-tumoral heterogeneity observed between tumors (Figure 1A and Figure 1B). When average staining intensity of IGF-1R/pIGF-1R and YAP/TAZ N:C ratios were treated as continuous variables, expression of IGF-1R and pIGF-1R were highly correlated (Pearson r correlation coefficient 0.690, *p* < 0.001). YAP and TAZ similarly correlated with each other (0.822, *p* < 0.001). Additionally, we identified a likely relationship between pIGF-1R and the YAP/TAZ pathway, as pIGF-1R was highly correlated with YAP and TAZ of 0.522 (*p* = 0.001). Weaker but statistically significant positive associations could be found between other pairs (Figure 1C).

Since the YAP/TAZ paralogs must bind TEAD (or other co-factors) and shuttle to the nucleus to exert their epigenetic effects, pathway activation can be measured using either the absolute expression of nuclear YAP/YAZ or the N:C ratios. Our patients’ observed N:C ratio ranged between 0.94 and 2.13 for YAP and 1.15 and 2.19 for TAZ. However, most patient values fell in much smaller interquartile ranges of 1.13 and 1.35 for YAP and 1.33 and 1.60 for TAZ (Figure 1D, left panel). IGF-1R nuclear intensity also had a narrow interquartile range compared to the total range. Conversely, we observed widely varied nuclear expression of pIGF-1R in patient samples (Figure 1D, right panel).

### Association of TMA confocal imaging with the histological characterization of disease

**Figure 2:**
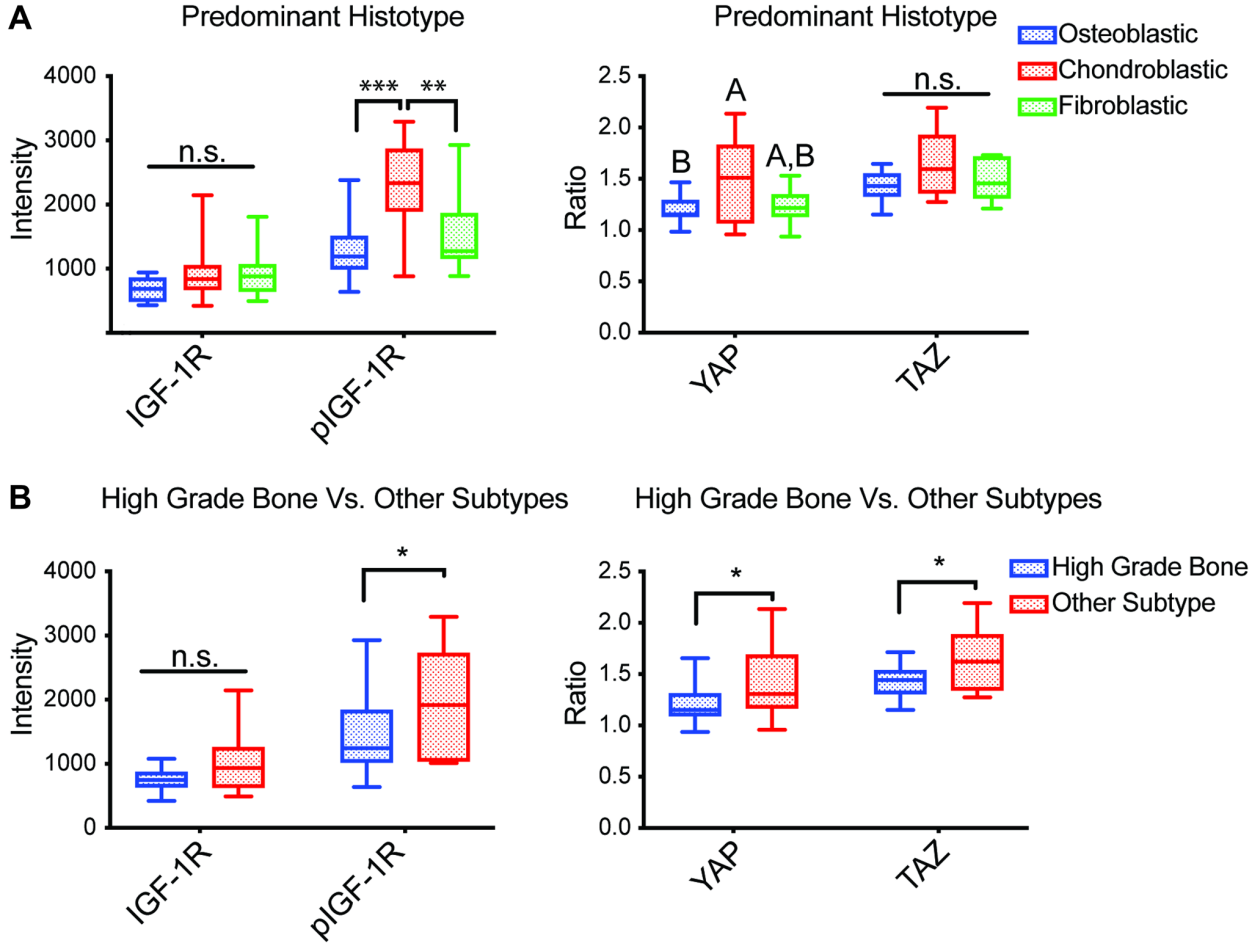
Associations between TMA staining and histopathological aspects of tumors. Average mean nuclear intensity of IGF-1R and pIGF-1R (Y1161) (*n* = 37) and average N:C ratio of YAP and TAZ (*n* = 36) in biopsies represented in a TMA subdivided by (**A**) predominant histotype of constituent cells [fibroblastic *n* = 10; osteoblastic *n* = 14; chondroblastic *n* = 7; other *n* = 4; not listed = 2], and (**B**) osteosarcoma subtypes compared to the high grade bone subtype [high-grade bone *n* = 27; other *n* = 10]. Box plots with inner line represent the interquartile range and median, where whiskers represent the range of observed values; Abbreviation: n.s.: no significance, differing letters and ^*^ *p* < 0.05, ^**^ *p* < 0.01, ^***^ *p* < 0.001, ANOVA with *post-hoc* Sidak test.

Since YAP/TAZ is an important role in directing MSCs lineage commitment toward mature connective tissues, we hypothesized their activity might associate with distinct osteosarcoma subtypes [21, 28]. Each tumor’s predominant histotype and the subtype were first determined by pathologists at MD Anderson Cancer Center that have bone sarcoma expertise. Notably, as OS tumors usually exhibit multi-lineage osteoblastic, chondroblastic, and fibroblastic differentiation, the relevant subclassification was determined by pathologists based upon their review of the entire tumor specimen. Next, we examined whether YAP/TAZ N:C ratios or pIGF-1R/IGF-1R expression levels differed by the OS histopathological subtype. Our findings indicate that YAP N:C ratio was highest among chondroblastic phenotype compared to other histotypes (Figure 2A, right panel). Increased YAP and TAZ N:C ratios were also observed in low- and intermediate-grade OS (Figure 2B, right panel).

Increased nuclear pIGF-1R was also associated with a chondroblastic histopathology and more likely to be seen in osteosarcoma subtypes other than high-grade bone (Figure 2, left panels). Interestingly, although increased pIGF-1R nuclear staining was associated with subtypes other than high-grade bone, IGF-1R nuclear staining intensity did not correlate with osteosarcoma subtype, possibly due to low sample sizes. However, when osteosarcoma subtypes were compared by specific subtype, the only statistical differences among the groups were an increased pIGF-1R nuclear stain and YAP N:C ratio of the myxoid subtype compared to high-grade bone (Supplementary Figure 1). Nuclear IGF-1R intensity and TAZ N:C ratios were not statistically different across the tumors with different predominant histotypes present in the osteosarcoma biopsies.

We also assessed if protein markers correlated with other clinical aspects of disease outcomes, as reported in other studies [29]. Interestingly, the YAP/TAZ N:C ratio and IGF-1R/pIGF-1R nuclear intensity did not appear to correlate with the primary vs. metastatic biopsy (Supplementary Figure 2A). Furthermore, they did not correlate with primary tumor location (Supplementary Figure 2B), the presence or absence of metastatic disease at diagnosis (Supplementary Figure 2C), or the emergence of metastases throughout a patient’s disease course (Supplementary Figure 2D).

### YAP/TAZ and pIGF-1R/IGF-1R staining and overall survival

Previous reports have associated high IGF-1R expression with advanced stage, lower overall survival, and distant metastasis. However, less is known about the impact of nuclear or phosphorylated IGF-1R in osteosarcoma [19, 30]. To correlate the nuclear staining and localization of the tumor markers with outcome measures, we found it useful to partition patients into groups with low, medium, and high YAP/TAZ N:C ratio or IGF-1R/pIGF-1R mean nuclear intensity. In our work, we stratified average values by grouping patients within the 1st quartile, interquartile range, or 4th quartile as “low,” “medium,” and “high,” respectively, for the four selected markers over the 37 patients represented in the TMA.

**Figure 3:**
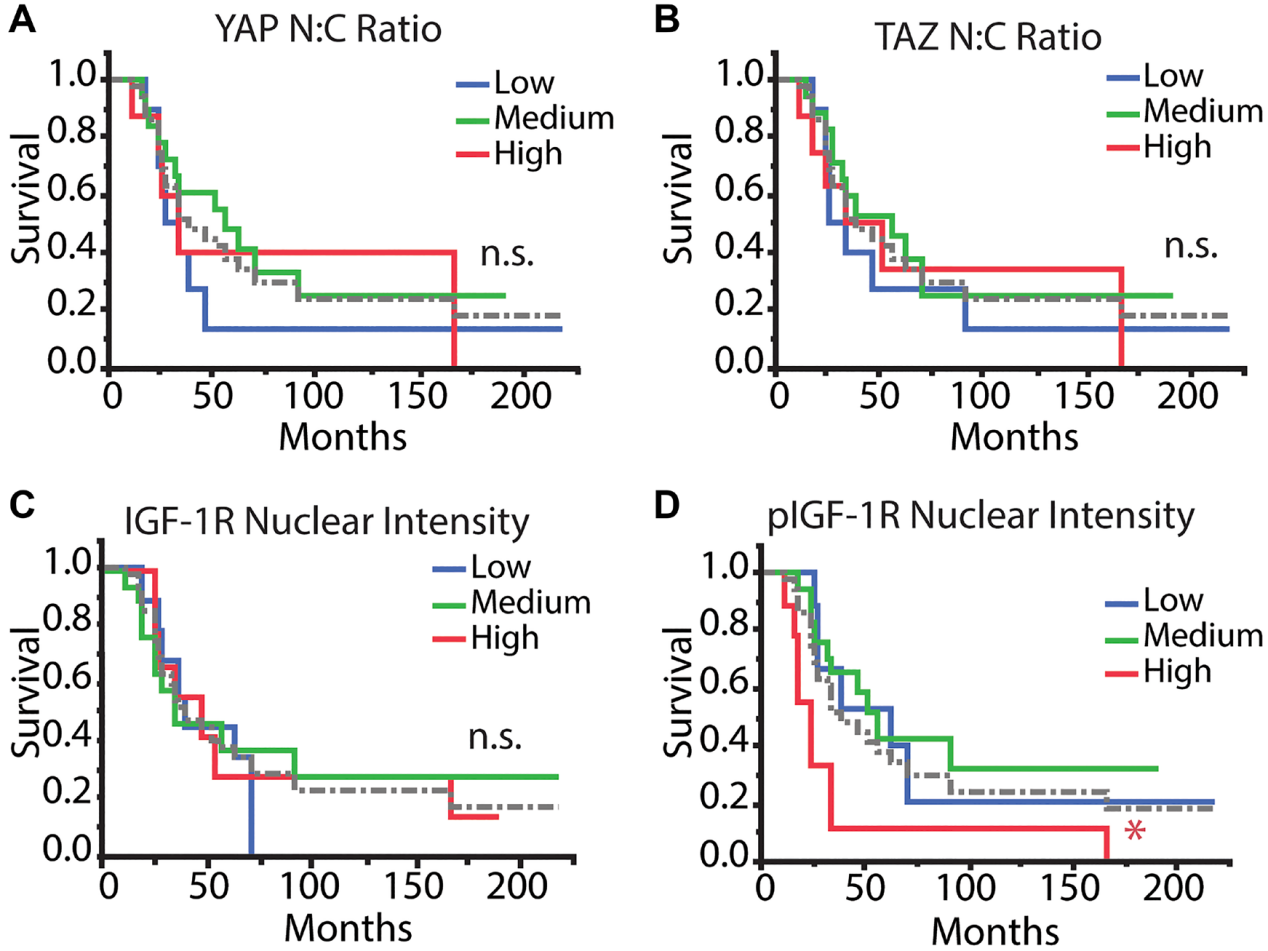
Kaplan–Meier survival analysis of tumor markers. Patients were stratified into low, medium, and high categories (1st quartile [*n* = 10 IGF-1R/pIGF-1R; *n* = 9 YAP/TAZ], interquartile range [*n* = 17 IGF-1R/pIGF-1R; *n* = 18 YAP/TAZ], 4th quartile [*n* = 10 IGF-1R/pIGF-1R; *n* = 9 YAP/TAZ] for each mean value across all patients, respectively) for each parameter and were analyzed with Kaplan–Meier analysis to determine differences in overall survival. Tumor markers included (**A**) YAP N:C ratio, (**B**) TAZ N:C ratio, (**C**) IGF-1R nuclear intensity, and (**D**) pIGF-1R nuclear intensity. The dotted gray line refers to the average of all patients. Log-rank test, *post-hoc*: Abbreviation: n.s.: no significance, ^*^ *p* < 0.05, ^**^ *p* < 0.01.

We assessed patients’ overall survival for correlations with patient characteristics, pathology assessments, or tumor marker values generated from confocal imaging of the TMA. Kaplan–Meier assessment yielded no statistical differences between YAP or TAZ N:C ratio or IGF-1R mean nuclear intensity and overall survival (Figure 3A–3C). Conversely, patients with high pIGF-1R mean nuclear intensity had worse overall survival (log-rank test done pairwise, *p* < 0.05) (Figure 3D).

**Figure 4:**
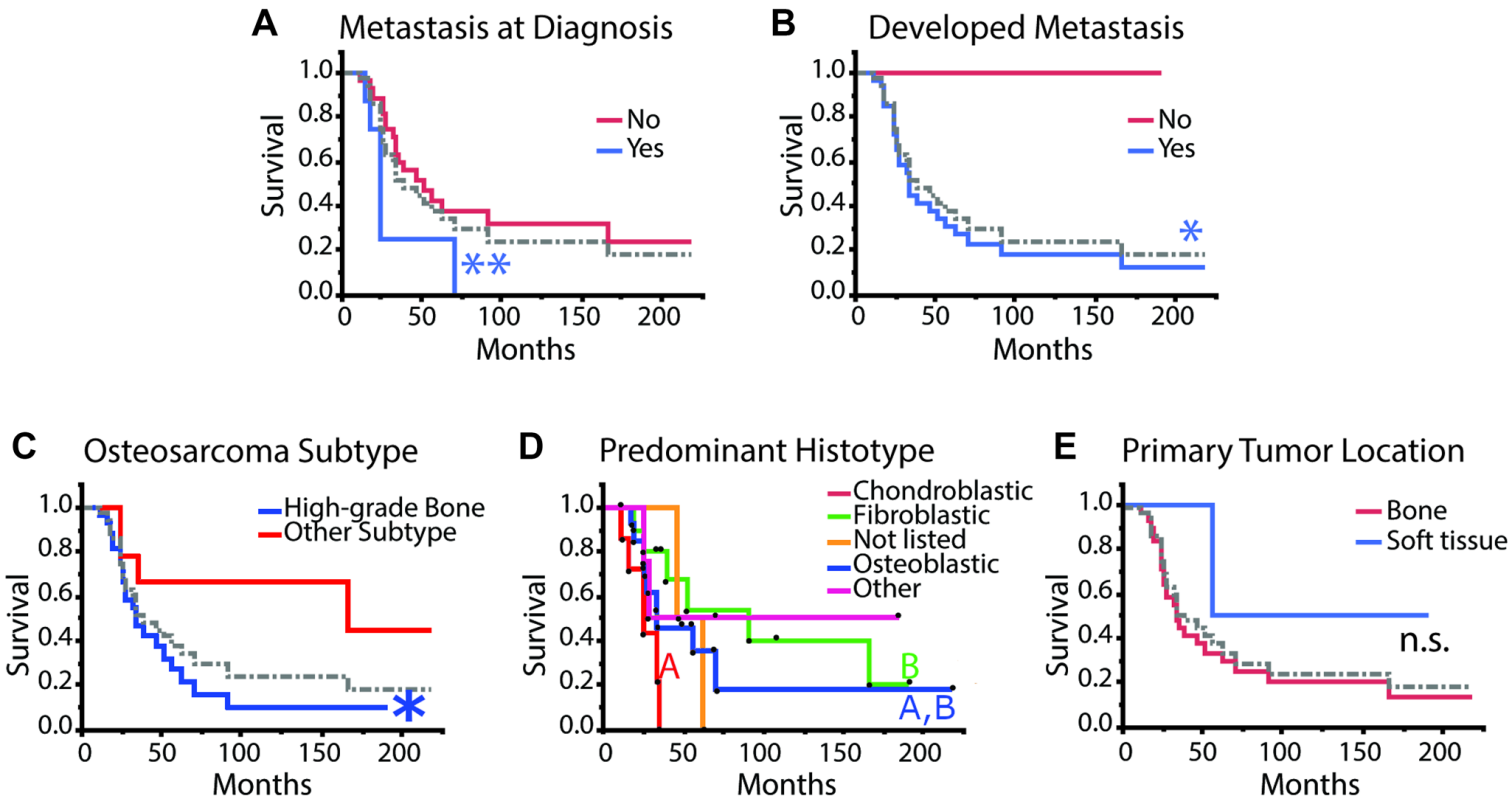
Kaplan–Meier survival analysis for clinical and histopathological aspects of disease. (**A**) metastasis at diagnosis: yes *n* = 8; no *n* = 28. (**B**) metastasis developed during disease course: yes *n* = 33; no *n* = 4. (**C**) osteosarcoma subtype: high-grade bone *n* = 27; other *n* = 10. (**D**) predominant histotype: excluding “not listed” *n* = 2 and “other” *n* = 4 and including “fibroblastic” *n* = 10; “osteoblastic” *n* = 14; “chondroblastic” *n* = 7. (**E**) primary tumor location: bone *n* = 33; soft tissue *n* = 4. Survival curves were compared using the log-rank test, and plots with more than two curves were compared pairwise except for “Predominant Histotype” where only the known histotype curves were compared excluding “not listed” and “other” and including “fibroblastic” *n* = 10; “osteoblastic” *n* = 14; “chondroblastic” *n* = 7. Black dots indicate individual patients that were censored (dot above curve) or died (dot below the curve). The dotted gray line refers to the average of all patients. Log-rank test, *post-hoc*: Abbreviation: n.s.: no significance, different colored letters and ^*^ *p* < 0.05, ^**^ *p* < 0.01.

To determine if pIGF-1R’s adverse survival impact was, in part, related to enhanced metastatic potential, we evaluated whether pIGF-1R (and other markers) occurred more commonly in patients who developed metastatic spread. As a first step, we reviewed whether known adverse clinical parameters in our data set were associated with poor patient outcomes. We correlated overall survival with metastasis at diagnosis and the development of clinically observable metastasis throughout the disease, which is a known negative prognostic indicator [31, 32]. As expected, patients diagnosed with metastatic disease at diagnosis or during their disease course had significantly worse overall survival (Figure 4A, 4B, log-rank test ^**^ *p* < 0.01 and ^*^ *p* < 0.05, respectively).

Consistent with published reports, the high-grade bone osteosarcoma subtype was associated with decreased overall survival (Figure 4C) [33]. Large studies have previously reported that the predominant histological subtype does not correlate with prognosis or outcomes in high-grade osteosarcoma, while a different group had reported that chondroblastic phenotypes might be associated with worse overall outcomes [34–36]. Interestingly, chondroblastic histotype was associated with worse outcomes than fibroblastic histotype, but neither of these phenotypes was statistically different from the osteoblastic histotype (Figure 4D). Primary tumor location did not seem to affect overall survival, which is consistent with previous studies (Figure 4E) [37]. We note that our study was not powered to detect a survival difference for extraskeletal osteosarcoma, given that this scenario is exceedingly rare.

Given the complexity and potential interactions between variables in our patient sample set, we developed a proportional hazards (Cox) model using *a priori* selected variables. Tumor markers were fit as categorical variables within the Kaplan–Meier analysis. Possibly due to the small sample size (*n* = 37), calculated risk ratios were considerable and unreliable (calculated *p* values for the Wald tests and hazard ratios with 95% CIs are available in Supplementary Table 1). To avoid overfitting our sample data, we employed the Bayesian information criterion (BIC) to select variables suitable for our Cox model that correlated with survival time in those patients who died over the observation period. This approach selected pIGF-1R mean nuclear intensity, YAP N:C ratio, osteosarcoma subtype, and predominant histotype as the variables most potentially predictive of time to death in the osteosarcoma patients represented in the TMA.

After fitting a Cox model to these four variables, Wald test *p*-values indicated that the effects of pIGF-1R mean nuclear intensity and YAP N:C ratio were significant (*p* = 0.0023 and *p* = 0.0224, respectively, Table 2). High pIGF-1R had increased risk ratios of 23.36 (95% CI: 3.83–154.72) and 25.22 (95% CI: 3.67–206.37) compared to medium and low pIGF-1R, respectively. There was no significant difference between medium and low pIGF-1R. The adverse prognostic effect of pIGF-1R upon survival was consistent with the Kaplan–Meier survival analysis. Conversely, it was low YAP N:C ratio compared to a medium N:C ratio that was associated with a higher risk ratio of 7.38 (95% CI: 1.81–35.42; *p* < 0.01). Lower YAP N:C ratio trended with an increased risk compared to high YAP N:C of 5.03 but was not statistically significant likely due to small sample size (95% CI: 0.58–76.15; *p* = 0.16). Calculated risk ratios, confidence intervals, and *p*-values for various pairs of osteosarcoma subtype and predominant histotype can be viewed in Supplementary Table 2, although the overall effect did not appear to be significant based on Wald tests for these variables.

**Table 2:** Proportional hazards model featuring variables selected with the Bayesian Information Criterion (BIC)

**Effect Wald Tests**					
**Source**			***p*-value **		
pIGF-1R Mean Nuclear Intensity			0.0023^*^		
YAP N:C Ratio			0.0224^*^		
Osteosarcoma Subtype			0.1689		
Predominant Histotype			0.1104		
**Risk Ratios**					
**Risk Ratios for pIGF-1R Mean Nuclear Intensity**					
**Level 1**	**Level 2**	**Risk Ratio**	***p*-value **	**Lower 95%**	**Upper 95%**
High	Low	25.22	0.0009^*^	206.37	3.67
Medium	Low	1.08	0.9021	0.33	3.94
High	Medium	23.36	0.0008^*^	3.83	154.72
**Risk Ratios for YAP N:C Ratio**					
**Level 1**	**Level 2**	**Risk Ratio**	***p*-value **	**Lower 95%**	**Upper 95%**
Low	High	5.03	0.1568	0.58	76.15
Low	Medium	7.38	0.0050^*^	1.81	35.43
High	Medium	1.47	0.7678	0.11	16.81

The fitted Cox models and the Kaplan–Meier survival curves indicated that YAP and pIGF-1R might be useful prognostic indicators in osteosarcoma. Because there is such a wide range of phenotypic variability in YAP localization and IGF-1R activation among patients, these indicators may be useful in stratifying or selecting patients for YAP/TAZ or IGF-1R targeted therapy.

## DISCUSSION

The rarity and heterogeneous intratumoral characteristics of osteosarcoma constitute significant hurdles for the development of prognostic biomarkers [1]. The advent of TMA technology has afforded cancer researchers the ability to examine multiple tissues from multiple patients. TMAs allow researchers to treat each sample simultaneously with stains and antibodies that can reduce variability between samples. The use of tissue microarrays has primarily been in studies focused on immunohistochemistry. Here we describe the use of quantitative image analyses for staining intensity and localization from confocal microscopy to evaluate TMAs, also reported in other studies [38].

In this work, we probed TMAs of core biopsies of osteosarcoma in patients with known outcomes to investigate the relationship between nuclear IGF-1R localization/activation and YAP/TAZ nuclear to cytoplasmic ratios. Usually, the IGF-1R/mTOR pathway facilitates the growth of organs, the proliferation of cells, and the development of many tissue types [39]. However, in osteosarcoma, the IGF-1R/mTOR pathway has been implicated as a driver of anti-apoptotic signals and aberrant growth in many cancers making the pathway an attractive target for potential therapies [40, 41].

The Hippo pathway effectors, YAP and TAZ, have been extensively described as transducers of mechanical inputs such as growth substrate stiffness, actin cytoskeletal tension, and cell shape [21, 22, 42]. Upon stimulation of the Hippo pathway in environments that confine cell growth or with low substrate stiffness, YAP and TAZ are phosphorylated and confined to the cytosol for degradation. This process helps regulate organ size in healthy tissues. Recent preclinical models in our lab and others have shown that increased cell spreading in less stiff 3D microenvironments promote nuclear localization of YAP/TAZ, highlighting a complex regulation of this mechanosensitive pathway [23, 24]. In osteosarcoma, YAP levels are upregulated in about 80% of tumors, and YAP/TAZ signaling is involved in tumor initiation, propagation, and metastasis [43–45]. This data suggests that decreased bone stiffness due to lytic osteosarcoma lesions may increase cell spreading and facilitate nuclear YAP/TAZ localization, thus contributing to pathogenesis.

Given the functions of YAP/TAZ and the IGF-1R/mTOR cascade in stimulating proliferation and growth, it is unsurprising that studies have revealed the potentiation of the mTOR pathway by YAP [46, 47]. However, to our knowledge, our study is the first to demonstrate this relationship in osteosarcoma patient biopsies. In patient samples, there was a wide variation of nuclear (i.e., active) IGF-1R, as evidenced by the distribution of mean intensities of samples stained for pIGF-1R. Kaplan–Meier survival analysis indicated that high nuclear pIGF-1R was associated with worse outcomes. Moreover, a selected Cox model fit to our data confirmed an increased hazard ratio for death for patients with progressively higher levels of nuclear pIGF-1R. Both statistical models corroborated previous studies that implicate IGF-1R/mTOR in aggressive osteosarcoma [19, 30, 48]. Indeed, these results suggest that IGF-1R or downstream signaling proteins may be attractive targets for therapy in osteosarcoma.

When tested in the clinic, IGF/PI3K/mTOR-directed therapies have yielded mixed results. This has led some groups to hypothesize that patient stratification based on IGF-1R expression may be necessary to select appropriate patients for targeted therapy [18, 19]. It is also plausible that single-agent IGF-1R-directed monoclonal antibodies and small molecule inhibitors are not effective enough to fully abrogate downstream mTOR signaling, as demonstrated in Ewing sarcoma preclinical models [49, 50].

Analysis of the TMA revealed consistent expression of YAP/TAZ N:C ratio across patient samples. A potential weakness of our analysis is that the biopsy specimens analyzed in this study were collected post-treatment from variable sites, as indicated in Table 1 and Supplementary Figure 2A. While prior studies assessed the relationship between nuclear or total YAP/TAZ and clinical outcome, we hypothesized that the N:C ratio of mean intensities for cells in each biopsy would serve as a proxy measure of YAP/TAZ activation [26, 43]. This approach allowed us to compare ratios of protein localization across tumor biopsies and determine the extent to which YAP/TAZ function is associated with cancer pathogenesis in multiple tumor types [25]. While univariate Kaplan–Meier survival analysis did not glean any statically significant association between YAP or TAZ N:C ratio and overall survival, a selected Cox model suggested that a low YAP N:C ratio is associated with decreased overall survival but only compared to the medium N:C ratio group not the high N:C ratio group. This result suggests that average values for YAP and TAZ N:C ratios may have prognostic value.

Our research identifies a potentially clinically important relationship between the Hippo pathway and IGF-1R/PI3K/mTOR pathway signaling. Other studies have shown that nuclear YAP and TAZ potentiates the signaling in the canonical IGF-1R/mTOR cascade as a potential mechanistic reason for this relationship [51, 52]. Though single-agent therapies against IGF-1R or mTOR have proved ineffective in osteosarcoma, the ability to co-target these pathways using novel biologically-targeted agents would be expected to yield synergistic anti-cancer activity, as has been observed in preclinical bone sarcoma models [53].

While sole targeting of the IGF/PI3K/mTOR cascade has had limited success in early phase osteosarcoma trials, our study suggests that nuclear pIGF-1R might serve as a prognostic biomarker to identify osteosarcoma patients that have an especially poor prognosis. Of course, prospective studies will be required to determine if patient selection, based on their nuclear pIGF-1R status, correlates with clinical response to IGF/PI3K/mTOR-directed therapies. Presently, most pharmaceutical companies have stopped the development of their IGF1 or IGF-1R-targeted drug candidates. This leaves only PI3K or mTOR as potential targets within this pathway. Given the likely pathway crosstalk between YAP/TAZ and IGF/PI3K/mTOR, one might hypothesize that a dual-targeting approach will have synergistic antineoplastic activity. This remains to be explored now that second-generation inhibitors of YAP1 (NCT04659096) and TEAD have reached the clinic.

## MATERIALS AND METHODS

### Study design

To establish our model’s clinical relevance, we used available osteosarcoma TMA samples consisting of two slides representing sequential tumor sections from 37 patients treated at The University of Texas MD Anderson Cancer Center. The TMA slide stained for YAP/TAZ lost one patient sample during preparation. When duplicate patient samples present on the slide, their results were averaged to give the tumor staining scores. The algorithms used to evaluate intensities in TMA samples were applied by a researcher blinded to the patients’ outcomes. Patient outcomes, demographics, and staging were compiled separately and not available to the researchers evaluating images of de-identified patient samples before analysis was complete.

### Statistical analyses

All statistics were performed using JMP Pro 14 or GraphPad Prism. Data are displayed as means or medians with standard deviations or as box blots indicating quartile ranges, as indicated in figure captions. Patient sample staining values were compared between groups using paired *t*-tests or one-way ANOVA with *post-hoc* Tukey’s HSD (if over two groups) or Sidak correction (2 groups) as described in figure captions with ^*^ *p* < 0.05, ^**^ *p* < 0.01, ^#^ *p* ≤ 0.001.

For Kaplan–Meier curve analyses and initial proportional hazards model, variables were selected *a priori*. The log-rank test was used to compare groups in Kaplan–Meier curves and done pairwise when three or more groups were present as indicated. To avoid overfitting our proportional hazards model to a small sample size, we selected patient variables for predicting death during the observation period using the Bayesian information criterion (BIC) method in JMP Pro 14. For completeness, we provide results of Cox models that were fit to a variable selected *a priori* and variables selected by the BIC.

### Preparation of tissue microarray

All aspects of our research were performed by recognized ethical guidelines (e.g., Declaration of Helsinki, Belmont Report). A TMA was constructed from archival surgical pathology materials comprising 38 formalin-fixed, paraffin-embedded tissues from 37 patients. Areas of viable tumor were selected by pathologist review of whole slide H&E-stained sections. Selected areas were punched and transferred, in duplicate, to a recipient block using an ATA-100 Advanced Tissue Arrayer (Chemicon International). All human specimens were utilized under an Institutional Review Board-approved research protocol allowing for the retrospective sampling and analysis of existing archival materials collected in the course of normal patient care. Immunofluorescence studies were performed using an antigen retrieval microwave (Biogenex) and manual staining with antibodies and stains described below.

### Immunofluorescence staining for confocal microscopy

Samples were then washed three times in 1× PBS on a shaker for 5 min before being blocked with blocking buffer (1 × PBS, 5% normal goat serum, 0.3% Triton^™^ X-100) for 60 min. Primary antibodies were then diluted 1:100 in antibody dilution buffer (1 × PBS, 1% BSA, 0.3% Triton^™^ X-100), and samples were incubated in primary antibody overnight at 4°C. Primary antibodies used were targeted to YAP (Santa Cruz, sc-271134), TAZ (Abcam, ab-84927), IGF-1R (Santa Cruz, sc-461), and pIGF-1R (Santa Cruz, sc-101703). Samples were then washed three times with 1 × PBS before incubating with secondary antibody (diluted 1:500, Cell Signaling Technology) and phalloidin conjugated to iFluor 488 (Abcam, diluted 1:1000, only in YAP/TAZ TMA) in antibody dilution buffer for 1.5 hours in the dark at room temperature. After three washes, cells were incubated for 10 min in a 1:5000 dilution of a 1 mg/mL Hoechst 33342 stock solution (ThermoFisher). Samples were then rinsed once with PBS. Coverslips were mounted onto slides using Prolong^®^ Gold Antifade Reagent (Cell Signaling Technology).

Because all samples were on one slide, the same power for each wavelength laser illumination was used for each sample to directly compare patient markers across samples. Individual tumor samples were stained for tumor markers of interest and nuclei and imaged using 20× objective by stitching images in a two-by-two image grid with 10% overlap to create one large image file for each tumor sample.

### Quantitative image analysis

All quantitative imaging analysis was done with IMARIS software (V8.6, Bitplane) licensed to Rice University. For YAP and TAZ, all areas staining positive for the Hoechst stain in the blue channel were considered nuclear domains. All areas staining positive for YAP in the red channel but not staining positive for nuclei in the blue channel were considered cytoplasmic domains. Mean nuclear and cytoplasmic intensity values for markers in the domains were identified by the IMARIS “cells” algorithm were then averaged after weighting each distinct identified domain by total area. Because individual cells were not able to be segmented in the highly cellular sections, we weighted each identified nuclear or cytoplasmic domain by area and averaged by the total area of the nuclear or cytoplasmic domain. In cases where the TMA contained two or more samples from the same patient, values were averaged between these two sections for further analysis. With this technique, average nuclear intensity and average cytoplasmic intensity for each tumor sample across the entire image were generated using the following formula:


$$N\text{:}C\;ratio=\frac{\frac{Mean\;Signal\;Intensity_{nuclear\;domain}\times Area_{nuclear\;domain}}{\sum_{i=1}^{n}Area_{nuclear\;domain}}}{\frac{Mean\;Signal\;Intensity_{cytoplasmic\;domain}\times Area_{cytoplasmic\;domain}}{\sum_{i=1}^{n}Area_{cytoplasmic\;domain}}}$$

Where *n* is the total number of nuclear or cytoplasmic domains identified in each patient sample.

For TMA stained for IGF-1R and pIGF-1R, individual nuclei were identified by distinct areas staining positive for the Hoechst stain in the blue channel. Values for mean nuclear intensities for the entire sample were then calculated by averaging the nuclear intensity of IGF-1R/pIGF-1R signals colocalizing with the blue channel. Each mean nuclear value was weighted by the nuclear area and averaged for each patient in the following formula:


$$Mean\;Nuclear\;Intensity=\frac{Mean\;Signal\;Intensity_{nuclear\;domain}\times Area_{nuclear\;domain}}{\sum_{i=1}^{n}Area_{nuclear\;domain}}$$

Where *n* is the total number of nuclear domains identified in all samples from the same patient.

## CONCLUSIONS

In this study, we demonstrated a correlation between nuclear IGF-1R and the Hippo pathway effectors, YAP and TAZ staining in human osteosarcoma. Further, we show that high nuclear-phosphorylated IGF-1R and low YAP N:C ratio are potentially negative prognostic indicators for overall survival in osteosarcoma. While histotype-specific YAP/TAZ activity must be confirmed in a larger dataset, our results imply a potential need for patient selection in trials directed at the IGF/PI3K/mTOR or Hippo pathways. Given the rarity of osteosarcoma, clinical validation of our results will almost certainly require the active participation of national and international high-volume cancer centers.

## SUPPLEMENTARY MATERIALS


