# Supplementary material for: Correlation of nuclear pIGF-1R/IGF-1R and YAP/TAZ in a tissue microarray with outcomes in osteosarcoma patients
Source: Oncotarget. 2022 Mar 9;13:521–33. doi: 10.18632/oncotarget.28215 (PMC8906536; doi:10.18632/oncotarget.28215)
Supplement: Supplementary file 1 [file oncotarget-13-28215-s001.pdf]

Correlation of nuclear pIGF-1R/IGF-1R and YAP/TAZ in a tissue microarray with outcomes in osteosarcoma patients

SUPPLEMENTARY MATERIALS

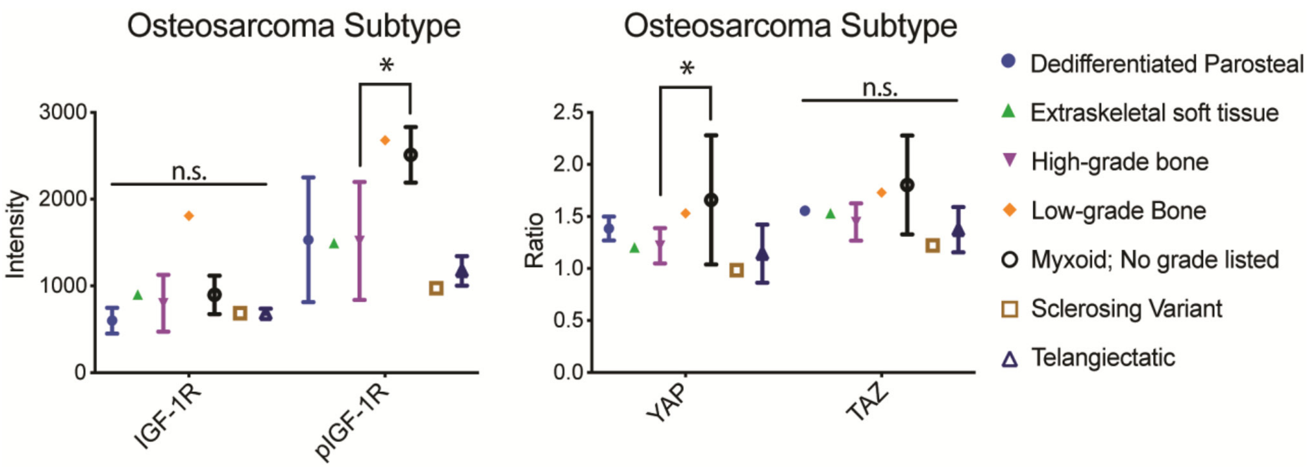

Supplementary Figure 1: Associations of TMA staining with osteosarcoma subtype. Average mean nuclear intensity of IGF-1R and pIGF-1R ($n = 37$) and average N:C ratio of YAP and TAZ ($n = 36$) in biopsies taken from primary, metastatic, or from locally recurring tumors from osteosarcoma represented in a TMA analyzed for associations with osteosarcoma subtype. Center line indicates the median value for subtype and the whiskers represent interquartile range of observed values. High-grade bone $n = 27$; dedifferentiated parosteal $n = 2$; telangiectatic $n = 2$; extraskeletal soft tissue $n = 1$; Myxoid $n = 3$; low-grade bone $n = 1$, sclerosing variant $n = 1$. ANOVA with post-hoc Sidak test.: Abbreviation: n.s.: no significance, $^{*}p < 0.05$, $^{**}p < 0.01$, $^{***}p < 0.001$.

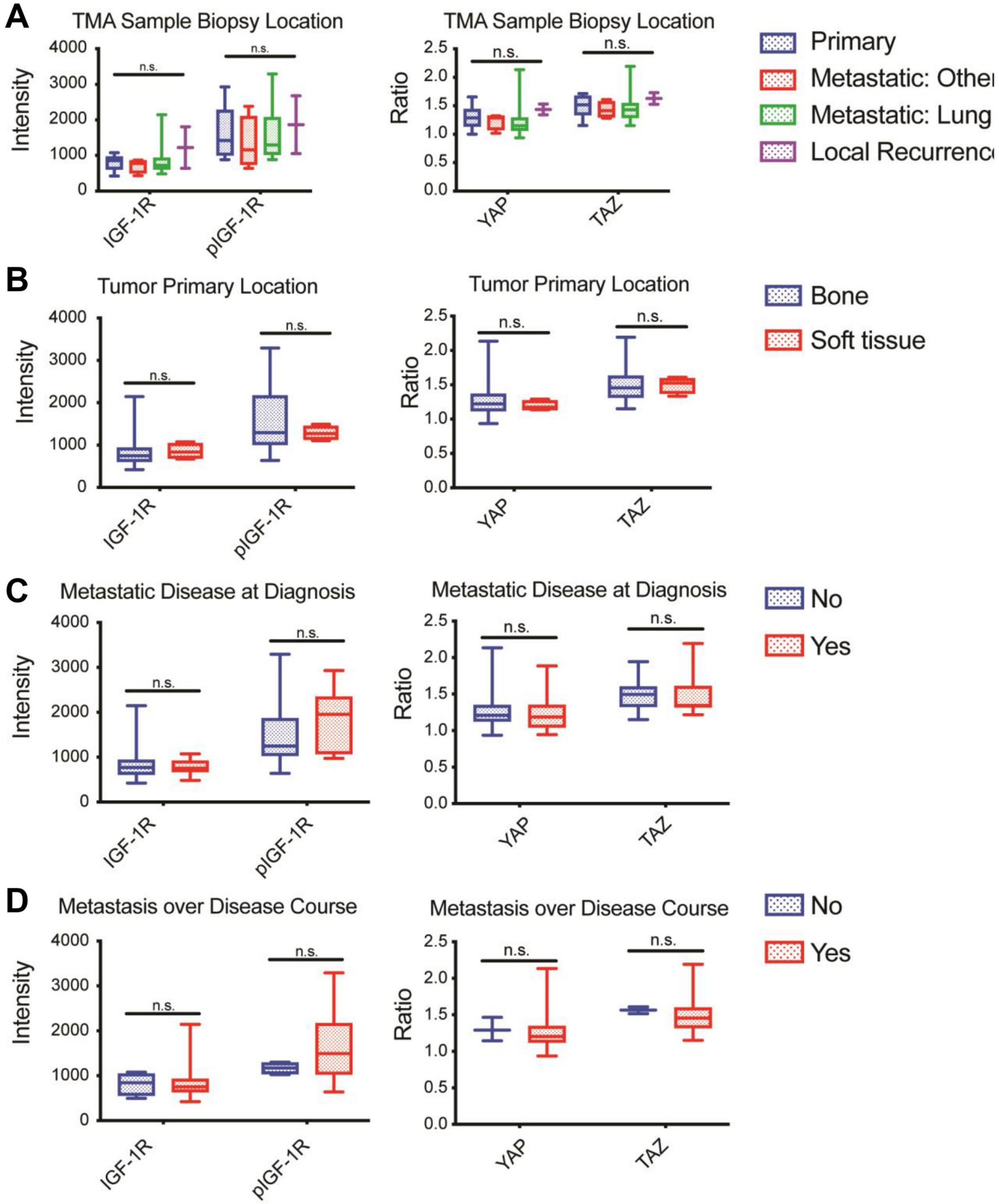

Supplementary Figure 2: Associations of TMA staining with biopsy location and clinical aspects of the disease. Average mean nuclear intensity of IGF-1R and pIGF-1R ($n = 37$) and average N:C ratio of YAP and TAZ ($n = 36$) in biopsies taken from primary, metastatic, or from locally recurring tumors from osteosarcoma represented in a TMA analyzed for associations with (**A**) biopsy location [primary $n = 10$; metastatic lung $n = 21$; primary $n = 4$; metastatic other site $n = 2$], (**B**) tumor primary location [bone $n = 33$; soft tissue $n = 4$], (**C**) whether the patient had clinically detectable metastases at diagnosis [yes $n = 8$; no $n = 28$], and (**D**) whether the patient developed metastases over the course of disease [yes $n = 33$; no $n = 4$]. Box plots with inner line represent the interquartile range and median, where whiskers represent the range of observed values; ANOVA with post-hoc Sidak test.: Abbreviation: n.s.: no significance, $^{*}p < 0.05$, $^{**}p < 0.01$, $^{***}p < 0.001$.

Supplementary Table 1: Risk ratios for YAP, TAZ, IGF-1R, and pIGF-1R biomarkers

Effect Wald Tests

Source	*P* value
IGF-1R Mean Nuclear Intensity	**0.0241***
pIGF-1R Mean Nuclear Intensity	*0.0006**
YAP N:C Ratio	*0.0074**
TAZ N:C Ratio	0.0778
Metastatic Disease at Diagnosis	**0.0340***
Osteosarcoma Subtype	0.1062
Predominant Histotype	**0.0428***
Skeletal or Extraskeletal	0.0533

Risk Ratios

Risk Ratios for IGF-1R Mean Nuclear Intensity

Level 1	Level 2	Risk Ratio	*P* value	Lower 95%	Upper 95%
Low	High	32.3323	0.0779	0.6294	1034.4292
Low	Medium	2.2690	0.5396	0.1044	22.1898
High	Medium	0.0702	*0.0027**	0.0082	0.4121

Risk Ratios for pIGF-1R Mean Nuclear Intensity

Level 1	Level 2	Risk Ratio	*P* value	Lower 95%	Upper 95%
Low	High	0.0002	*0.0011**	0.0000	0.0247
Low	Medium	0.0058	**0.0372***	0.0001	0.7090
High	Medium	34.8544	*0.0009**	4.1906	404.0291

Risk Ratios for YAP N:C Ratio

Level 1	Level 2	Risk Ratio	*P* value	Lower 95%	Upper 95%
Low	High	0.4432	0.5308	0.0355	6.6835
Low	Medium	111.4736	*0.0003**	7.6122	3603.6885
High	Medium	251.5273	*0.0076**	4.7276	17682.8730

Risk Ratios for TAZ N:C Ratio

Level 1	Level 2	Risk Ratio	*P* value	Lower 95%	Upper 95%
Low	High	1.6962	0.6174	0.2048	15.4052
Low	Medium	0.1585	0.0907	0.0112	1.3177
High	Medium	0.0935	**0.0171***	0.0096	0.6689

Risk Ratios for Metastatic Disease at Diagnosis

Level 1	Level 2	Risk Ratio	*P* value	Lower 95%	Upper 95%
Yes	No	6.1190	**0.0347***	1.1437	35.5930

Risk Ratios for Osteosarcoma Subtype

Level 1	Level 2	Risk Ratio	*P* value	Lower 95%	Upper 95%
Dedifferentiated Parosteal	Dedifferentiated Parosteal; High Grade	528.1289	0.9999	0.0000	.
Dedifferentiated Parosteal	Extraskeletal; Soft Tissue	0.0131	1.0000	0.0000	.
Dedifferentiated Parosteal; High Grade	Extraskeletal; Soft Tissue	0.0000	0.9999	0.0000	.
Dedifferentiated Parosteal	Bone: High Grade	0.0000	*0.0032**	0.0000	.
Dedifferentiated Parosteal; High Grade	Bone: High Grade	0.0000	*0.0005**	0.0000	.
Extraskeletal; Soft Tissue	Bone: High Grade	0.0000	0.3742	0.0000	.
Dedifferentiated Parosteal	Bone: Low Grade	0.0000	0.8318	0.0000	.
Dedifferentiated Parosteal; High Grade	Bone: Low Grade	0.0000	0.0509	0.0000	.
Extraskeletal; Soft Tissue	Bone: Low Grade	0.0000	0.9845	0.0000	.
Bone: High Grade	Bone: Low Grade	2710.2899	*0.0003**	11.2060	655511.3700
Dedifferentiated Parosteal	Myxoid	0.0000	0.2865	0.0000	.
Dedifferentiated Parosteal; High Grade	Myxoid	0.0000	*0.0094**	0.0000	.
Extraskeletal; Soft Tissue	Myxoid	0.0000	0.8789	0.0000	.
Bone: High Grade	Myxoid	42.8674	**0.0115***	1.4967	1227.7701

Bone: Low Grade	Myxoid	0.0158	0.1505	0.0000	5.8551
Dedifferentiated Parosteal	Sclerosing Variant	0.1886	1.0000	0.0000	.
Dedifferentiated Parosteal; High Grade	Sclerosing Variant	0.0004	0.9997	0.0000	.
Extraskeletal; Soft Tissue	Sclerosing Variant	14.4111	1.0000	0.0000	.
Bone: High Grade	Sclerosing Variant	32060000000	**0.0398**[*]	0.0000	.
Bone: Low Grade	Sclerosing Variant	11830776	0.8785	0.0000	.
Myxoid	Sclerosing Variant	748000319.	0.4370	0.0000	.
Dedifferentiated Parosteal	Telangiectatic	0.0000	0.9374	0.0000	.
Dedifferentiated Parosteal; High Grade	Telangiectatic	0.0000	0.1416	0.0000	.
Extraskeletal; Soft Tissue	Telangiectatic	0.0000	0.9944	0.0000	.
Bone: High Grade	Telangiectatic	20851.0680	**0.0140**[*]	12.6308	34421131.0000
Bone: Low Grade	Telangiectatic	7.6933	0.5445	0.0118	5033.6723
Myxoid	Telangiectatic	486.4084	0.0882	0.3857	613471.3200
Sclerosing Variant	Telangiectatic	0.0000	0.9557	0.0000	.

Risk Ratios for Predominant Histotype

**Level 1**	**Level 2**	**Risk Ratio**	***P* value**	**Lower 95%**	**Upper 95%**
Chondroblastic	Fibroblastic	0.2627	0.2570	0.0269	2.5635
Chondroblastic	Not listed	0.1030	0.1523	0.0047	2.2500
Fibroblastic	Not listed	0.3920	0.4627	0.0336	4.5704
Chondroblastic	Osteoblastic	0.0394	**0.0115**[*]	0.0026	0.5929
Fibroblastic	Osteoblastic	0.1500	*0.0078*[*]	0.0336	0.6688
Not listed	Osteoblastic	0.3827	0.4835	0.0244	6.0068
Chondroblastic	Other	0.0001	**0.0147**[*]	0.0000	0.1183
Fibroblastic	Other	0.0005	*0.0075*[*]	0.0000	0.1028
Not listed	Other	0.0013	**0.0200**[*]	0.0000	0.3865
Osteoblastic	Other	0.0035	**0.0347**[*]	0.0000	0.4086

Risk Ratios for Skeletal or Extraskeletal

**Level 1**	**Level 2**	**Risk Ratio**	***P* value**	**Lower 95%**	**Upper 95%**
Bone	Soft tissue	16.8238	**0.0282**[*]	1.3227	554.6511

Supplementary Table 2: Risk ratios for osteosarcoma subtypes

Level 1	Level 2	Risk Ratio	*P* Value	Lower 95%	Upper 95%
**Risk Ratios for Osteosarcoma Subtype**					
**Level 1**	**Level 2**	**Risk Ratio**	***P* Value**	**Lower 95%**	**Upper 95%**
Dedifferentiated Parosteal; High Grade	Dedifferentiated Parosteal	0.1454312	1	0	.
Extraskeletal; Soft Tissue	Dedifferentiated Parosteal	1.4717368	1	0	.
Extraskeletal; Soft Tissue	Dedifferentiated Parosteal; High Grade	10.119814	1	0	.
Bone; High Grade	Dedifferentiated Parosteal	4.96E+09	0.1447	0	.
Bone; High Grade	Dedifferentiated Parosteal; High Grade	3.41E+10	**0.0143***	0	.
Bone; High Grade	Extraskeletal; Soft Tissue	3.37E+09	0.2035	0	.
Bone; Low Grade	Dedifferentiated Parosteal	111744582	0.7592	0	.
Bone; Low Grade	Dedifferentiated Parosteal; High Grade	768367251	0.3826	0	.
Bone; Low Grade	Extraskeletal; Soft Tissue	75927015	0.8419	0	.
Bone; Low Grade	Bone; High Grade	0.0225317	**0.0262***	0.0005359	0.9473154
Myxoid	Dedifferentiated Parosteal	442542669	0.6	0	.
Myxoid	Dedifferentiated Parosteal; High Grade	3.04E+09	0.1898	0	.
Myxoid	Extraskeletal; Soft Tissue	300694165	0.6985	0	.
Myxoid	Bone; High Grade	0.0892326	0.0571	0.0055473	1.4353684
Myxoid	Bone; Low Grade	3.9603054	0.6021	0.0210314	745.74451
Sclerosing Variant	Dedifferentiated Parosteal	0.1108691	1	0	.
Sclerosing Variant	Dedifferentiated Parosteal; High Grade	0.762347	1	0	.
Sclerosing Variant	Extraskeletal; Soft Tissue	0.0753321	1	0	.
Sclerosing Variant	Bone; High Grade	2.24E-11	*0.0003**	0	.
Sclerosing Variant	Bone; Low Grade	9.92E-10	0.3196	0	.
Sclerosing Variant	Myxoid	2.51E-10	0.0959	0	.
Telangiectatic	Dedifferentiated Parosteal	383968801	0.5962	0	.
Telangiectatic	Dedifferentiated Parosteal; High Grade	2.64E+09	0.129	0	.
Telangiectatic	Extraskeletal; Soft Tissue	260895019	0.7115	0	.
Telangiectatic	Bone; High Grade	0.077422	0.1311	0.0027771	2.1584544
Telangiectatic	Bone; Low Grade	3.436129	0.5896	0.037222	317.2041
Telangiectatic	Myxoid	0.8676424	0.9479	0.0122578	61.414232
Telangiectatic	Sclerosing Variant	3.46E+09	0.1323	0	.
**Risk Ratios for Predominant Histotype**					
**Level 1**	**Level 2**	**Risk Ratio**	***P* Value**	**Lower 95%**	**Upper 95%**
Chondroblastic	Fibroblastic	1.5093429	0.7647	0.1063786	21.415178
Chondroblastic	Not listed	1.0013466	0.9993	0.0562731	17.818358
Fibroblastic	Not listed	0.6634322	0.6657	0.1070028	4.1133699
Chondroblastic	Osteoblastic	0.3193135	0.3611	0.0229583	4.441138
Fibroblastic	Osteoblastic	0.211558	**0.0120***	0.0588888	0.7600217
Not listed	Osteoblastic	0.3188841	0.1681	0.0545035	1.8656992
Chondroblastic	Other	0.0612886	0.1533	0.0012912	2.9090637
Fibroblastic	Other	0.0406062	0.055	0.0025369	0.6499427
Not listed	Other	0.0612062	0.1039	0.0026431	1.4173245
Osteoblastic	Other	0.1919387	0.2406	0.0166468	2.2130695
